# Potential Relationship Between Ferroptosis and Pyroptosis in Myocardial Ischemia/Reperfusion Injury: Molecular Mechanisms and Therapeutic Targets

**DOI:** 10.31083/RCM50196

**Published:** 2026-07-14

**Authors:** Yuxin Zhu, Hongmei Zheng, Hongshi Li, Li Xue, Heng Cai, Chongzhe Pei

**Affiliations:** ^1^Department of Cardiology, Tianjin Medical University General Hospital, 300052 Tianjin, China; ^2^The First Clinical College, Tianjin Medical University, 300070 Tianjin, China

**Keywords:** myocardial ischemia/reperfusion injury, ferroptosis, pyroptosis, mechanisms, therapy, relationship

## Abstract

While restoring blood flow is the primary treatment for acute myocardial infarction (AMI), this process often triggers additional damage known as myocardial ischemia/reperfusion injury (MIRI). Recent studies have suggested that regulated cell death is a major contributor to cardiomyocyte injury during ischemia and subsequent reperfusion. Notably, emerging evidence has demonstrated that both ferroptosis and pyroptosis play important roles in the pathogenesis of MIRI. Ferroptosis is primarily driven by iron-dependent lipid peroxidation, whereas pyroptosis is characterized by inflammasome-mediated inflammatory cell death. However, these processes are typically studied independently, and their potential interactions and shared regulatory mechanisms remain unclear. In this review, we comprehensively summarize the molecular mechanisms underlying ferroptosis and pyroptosis in MIRI. We also discuss emerging evidence supporting the crosstalk between these two processes, with a particular focus on shared upstream triggers such as reactive oxygen species (ROS), mitochondrial dysfunction, and common regulatory proteins, including nuclear factor erythroid 2-related factor 2 (Nrf2), p53, and members of the high mobility group box (HMGB) family. Furthermore, we highlight current therapeutic strategies targeting ferroptosis and pyroptosis and explore the potential of dual-targeting approaches based on their shared signaling pathways. A deeper understanding of the interplay between these two forms of cell death may provide novel insights into the pathogenesis of MIRI and support the development of more effective cardioprotective strategies.

## 1. Introduction

Ischemic heart disease (IHD) is currently a top cause of death and poor health globally [1]. Its most common form is acute myocardial infarction (AMI). Currently, the most effective treatment for AMI patients is to restore blood flow quickly, typically through thrombolysis or percutaneous coronary intervention (PCI) [2]. However, restoring blood flow can induce additional myocardial injury, leading to severe arrhythmias and sudden death [3]. This phenomenon is known as myocardial ischemia/reperfusion injury (MIRI). The underlying pathology of MIRI is intricate and includes multiple interrelated mechanisms, such as oxidative stress, mitochondrial dysfunction, ionic imbalance and inflammatory responses. Increasing evidence suggests that regulated cell death is a key reason for cardiomyocyte injury during ischemia and reperfusion, thereby contributing to the progression of MIRI [4].

In recent years, two specific types of cell death, ferroptosis and pyroptosis, have gained significant attention in cardiovascular diseases [5,6]. Ferroptosis is an iron-dependent regulated cell death [7], whereas pyroptosis is an inflammatory type of death mediated by inflammasomes [8]. Table 1 summarizes the key characteristics of ferroptosis and pyroptosis. The progression of MIRI is driven by both processes. Interestingly, studies in other diseases such as epilepsy [9], heart failure [10], drug resistance [11], asthma and chronic obstructive pulmonary disease (COPD) [12] have shown a clear link between ferroptosis and pyroptosis. Nevertheless, in MIRI, the mechanisms underlying ferroptosis and pyroptosis, as well as therapeutic strategies targeting these two forms of cell death, are usually investigated independently. The direct experimental evidence simultaneously examining their mechanistic interplay in the heart, especially in MIRI, remains extremely limited. Therefore, the potential interaction and crosstalk between ferroptosis and pyroptosis in MIRI, together with their shared regulatory mechanisms, remain incompletely understood and are largely inferred from parallel mechanistic findings rather than direct cardiac evidence. In this review, we first summarize the fundamental molecular mechanisms of ferroptosis and pyroptosis in MIRI. However, we do not aim to merely reiterate the individual roles of ferroptosis or pyroptosis in MIRI. Instead, we provide an integrative and concept-driven synthesis of current evidence with three specific advances. First, we distinguish simple co-activation from potential direct mechanistic links between ferroptosis and pyroptosis, highlighting shared upstream triggers and feedback loops. Second, we incorporate emerging data on the temporal dynamics of ferroptosis, pyroptosis and their shared regulatory molecules during ischemia and reperfusion, thereby proposing a stage-dependent framework of their interaction. Third, we extend the discussion beyond cardiomyocytes to summarize intercellular crosstalk involving endothelial cells, immune cells, and inflammatory mediators, emphasizing how ferroptotic and pyroptotic signals may propagate across cell types within the ischemic heart. Finally, we evaluate current therapeutic strategies, focusing on the challenges of clinical translation and the potential of dual-targeting approaches. This comprehensive framework aims to provide new perspectives for future mechanistic investigations and therapeutic strategies targeting ferroptosis and pyroptosis in MIRI.

**Table 1.** **Characteristics of ferroptosis and pyroptosis**.

	Ferroptosis	Pyroptosis
Definition	Ferroptosis is an iron-dependent regulated cell death.	Pyroptosis is an inflammation-mediated regulated cell death.
Cell death type	Regulated, non-apoptotic	Regulated, inflammatory
Primary triggers	Iron overload, lipid peroxidation, failure of anti-oxidative defense system	DAMPs/PAMPs, ROS, ionic imbalance
Key mediators	GPX4, SLC7A11, ACSL4, ALOX15	NLRP3, Caspase-1, GSDMD
Mitochondrial involvement	mtROS generation, lipid peroxidation	mtROS and mtDNA-mediated inflammasome activation
Morphological changes	Mitochondrial shrinkage	Cell swelling and membrane rupture
Predominant phase in MIRI	Reperfusion phase	Reperfusion phase
Shared regulators	Nrf2, HMGB1, HMGB2, p53	
Crosstalk-related mechanisms	Oxidative stress, ROS accumulation, and mitochondrial dysfunction	

Abbreviation used: DAMPs, damage-associated molecular patterns; PAMPs, pathogen-associated molecular patterns; ROS, reactive oxygen species; GPX4, glutathione peroxidase 4; SLC7A11, solute carrier family 7 member 11; ACSL4, acyl-CoA synthetase long-chain family member 4; ALOX15, arachidonate 15-lipoxygenase; NLRP3, NOD-like receptor family pyrin domain containing 3; GSDMD, gasdermin D; mtROS, mitochondrial reactive oxygen species; MIRI, myocardial ischemia/reperfusion injury; Nrf2, nuclear factor erythroid 2-related factor 2; HMGB1, high mobility group box 1; HMGB2, high mobility group box 2.

## 2. Literature Review

We performed a targeted literature search using PubMed and Web of Science databases to identify relevant studies published from January 2020 to December 2025. The search terms included combinations of “myocardial ischemia/reperfusion injury”, “myocardial I/R injury”, “MIRI”, “ferroptosis”, “pyroptosis”, “programmed cell death”, “crosstalk”, “therapy”, “treatment”, and “therapeutic targets”. Inclusion criteria were as follows: (1) original experimental studies or high-quality review articles; (2) studies investigating ferroptosis and/or pyroptosis in the context of myocardial ischemia/reperfusion injury or closely related cardiac ischemic conditions; (3) studies providing mechanistic insights, molecular pathways, or regulatory factors relevant to ferroptosis, pyroptosis, or their potential crosstalk; (4) studies conducted in cardiomyocytes, cardiac endothelial cells, fibroblasts, macrophages, or in vivo cardiac ischemia/reperfusion models; (5) articles published in English. Exclusion criteria included: (1) studies unrelated to cardiac tissue or ischemia/reperfusion contexts; (2) studies focusing on other forms of cell death without relevance to ferroptosis or pyroptosis; (3) conference abstracts, editorials, letters, or non-peer-reviewed publications; (4) articles not written in English. Additional relevant articles were identified by manual screening of the reference lists of selected publications. This review was conducted as a narrative review rather than a systematic review.

## 3. Ferroptosis in MIRI

### 3.1 Definition and Molecular Mechanisms of Ferroptosis

Ferroptosis represents a distinct modality of regulated cell death driven by iron overload, lipid peroxidation and failure of the anti-oxidative defense system [13,14]. Iron is an important element, that plays crucial roles in the human body, including hemoglobin-mediated oxygen transport and enzymatic catalysis [15]. However, iron overload can activate the Fenton reaction, leading to the excessive production of reactive oxygen species (ROS) [16]. Physiologically, various intracellular membrane structures, such as the cell membrane, mitochondrial membrane, endoplasmic reticulum (ER) membrane and lysosomal membrane are rich in polyunsaturated fatty acids (PUFAs), which are highly susceptible to oxidation [17]. Excessive iron and ROS could oxidize PUFAs within these membranes, resulting in membrane damage. This damage compromises cellular homeostasis and ultimately leads to cell death. Among these organelles, lysosomes play a particularly important role in ferroptosis, as they serve as major storage sites for free Fe^2+^. Consequently, damage to the lysosomal membrane triggers the release of substantial quantities of Fe2+ into the cytoplasm, further aggravating ferroptosis. Under normal conditions, cells possess an endogenous antioxidant defense system. System Xc- imports cystine to support the production of glutathione (GSH). Glutathione peroxidase 4 (GPX4) reduces oxidative PUFAs to their non-oxidized forms using GSH as a cofactor [18]. Therefore, the dysfunction of the GPX4/GSH axis can trigger or mediate ferroptosis. Besides the classic GPX4-dependent ferroptotic pathway, researchers have also demonstrated other GPX4-independent pathways, such as SRY-box transcription factor 4 (SOX4)/death-associated protein kinase (DAPK) pathway [19], arachidonate 15-lipoxygenase (ALOX15)/15-hydroperoxyeicosatetraenoic acid (15-HpETE) pathway [20] and ataxia telangiectasia mutated (ATM)/p53 pathway [21].

### 3.2 Role and Mechanisms of Ferroptosis in MIRI

Recently, growing evidence has identified ferroptosis as a key contributor to cell death in MIRI [22]. Therefore, obtaining a clear understanding of the mechanisms underlying ferroptosis in MIRI is of great importance. Distinct from the ischemic period, the execution of ferroptosis is predominantly observed during the subsequent reperfusion stage [23]. When the occluded coronary artery is reopened, the rapid influx of oxygen-rich blood into the myocardium triggers a burst of ROS production. At the same time, the intracellular levels of Fe^2+ ^in cardiomyocytes are up-regulated [23], which can activate the Fenton reaction, further increasing ROS generation and thereby exacerbating ferroptosis. Moreover, GPX4, GSH and System Xc^-^ are all down-regulated upon reperfusion [24], resulting in the dysfunction of the antioxidant defense system and the induction of ferroptosis.

Miyamoto et al. [25] identified iron overload as a critical contributor to ferroptosis in cardiomyocytes. Under hypoxic or hypoxia/reoxygenation conditions, heme oxygenase-1 (HO-1) is up-regulated in cardiomyocytes. This leads to excessive iron accumulation and triggers ferroptosis within the ER. Zhao et al. [26] demonstrated that during the reperfusion phase, Zrt- and Irt-like protein 14 (ZIP14) expression is markedly increased, inducing iron-dependent lipid peroxidation within lysosomes, thereby aggravating cardiomyocyte damage. Additionally, Cai et al. [20] reported that the expression of ALOX15 increases after reperfusion in cardiomyocytes, especially in damaged myocardial regions, where it catalyzes the synthesis of 15-HpETE. This lipid peroxide facilitates the interaction between peroxisome proliferator-activated receptor gamma coactivator 1 alpha (PGC-1α) and the ubiquitin ligase ring finger protein 34 (RNF34), promoting the ubiquitin-mediated degradation of PGC-1α and leading to mitochondrial dysfunction. Abnormal mitochondrial function further accelerates lipid peroxidation and ferroptosis. A recent study revealed that hypoxia induces the lactylation of acyl-CoA synthetase long-chain family member 4 (ACSL4), a key promoter of ferroptosis, thereby enhancing its expression level and stability and aggravating ferroptosis [27]. Wang et al. [28] identified a novel molecular axis regulating ferroptosis in cardiomyocytes. During myocardial ischemia/reperfusion, the double-stranded DNA (dsDNA)/cyclic GMP-AMP synthase (cGAS)/stimulator of interferon genes (STING) pathway is activated. STING directly targets GPX4 and promotes its autophagic degradation by facilitating autophagosome-lysosome fusion, thereby intensifying ferroptosis and ischemia/reperfusion injury. Finally, the integrity of the solute carrier family 7 member 11 (SLC7A11)/GSH/GPX4 axis remains central to preventing injury. Chen et al. [29] confirmed that hypoxia/reoxygenation compromises this defense system by down-regulating SLC7A11, which depletes GSH and suppresses GPX4 activity, ultimately triggering cardiomyocyte death.

Some metabolic diseases can also modulate ferroptosis in MIRI. Zhang et al. [30] identified a relationship between hyperuricemia and ferroptosis in MIRI and revealed that the elevated levels of uric acid could induce excessive ROS production, the dysfunction of mitochondria and alterations in the expression of key ferroptosis-related proteins in cardiomyocytes. Hyperuricemia was shown to exacerbate ferroptosis by regulating nuclear receptor co-activator 4 (NCOA4)/ferritin heavy chain 1 (FTH1) and GSH/GPX4 pathways. Diabetes is another critical comorbidity that increases susceptibility to ferroptosis in MIRI. Huang et al. [31] found that following myocardial ischemia/reperfusion, diabetic rats exhibited more severe cardiac dysfunction than non-diabetic rats, accompanied by elevated glycolipid levels, increased creatine kinase-MB and apoptotic index, as well as up-regulated expression of nuclear receptor subfamily 1 group D member 1 (Rev-erbα) and ferroptosis-associated proteins in cardiomyocytes.

## 4. Pyroptosis in MIRI

### 4.1 Definition and Molecular Mechanisms of Pyroptosis

Pyroptosis is an inflammatory type of regulated cell death that relies on the formation of membrane pores. Unlike ferroptosis, it induces cellular swelling and plasma membrane rupture, leading to the release of inflammatory signals [32]. Generally, the innate immune system senses danger through two primary types of signals. They are damage-associated molecular patterns (DAMPs), like adenosine triphosphate (ATP), ROS and mitochondrial DNA (mtDNA), and pathogen-associated molecular patterns (PAMPs), such as lipopolysaccharide (LPS) and bacterial toxins [33]. These molecular patterns are capable of activating inflammasomes. Among various inflammasomes, the most essential mediator of pyroptosis is the NOD-like receptor family pyrin domain containing 3 (NLRP3) inflammasome, as it has been the most extensively investigated, and is deeply implicated in the pathogenesis of diverse cardiovascular pathologies [34]. The NLRP3 inflammasome is composed of NLRP3, apoptosis-associated speck-like protein containing a CARD (ASC) and pro-caspase-1 [35]. The accumulation of ROS and ionic imbalances like K^+ ^efflux and Ca^2+^ overload, accompanied by mitochondrial dysfunction and mtDNA release [36], collectively trigger NLRP3 inflammasome activation. This activation subsequently induces the processing of caspase-1. Once active, caspase-1 matures the inflammatory cytokines to produce interleukin 1 beta (IL-1β) and interleukin 18 (IL-18), and cleaves gasdermin D (GSDMD) into two fragments. The gasdermin D N-terminal (GSDMD-N) is the key mediator of pyroptosis. It moves to the cell membrane and punches non-selective pores. These pores destroy the cell’s balance, causing Na^+ ^and water to enter the cytoplasm. As a result, the cell swells and ruptures [37]. Meanwhile, IL-18 and IL-1β are released through these pores, along with other cell contents, which can trigger an immune response. Besides the NLRP3-dependent classical pathway, there are also some non-classical pathways inducing pyroptosis. For instance, in the caspase-4/5/11 pathway, LPS directly activates caspase-4/5 (in humans) or caspase-11 (in mice), which subsequently cleaves GSDMD, resulting in pyroptosis [38]. Moreover, the granzyme pathway and the caspase-3/8 pathway are both important contributors to pyroptosis [39].

### 4.2 Role and Mechanisms of Pyroptosis in MIRI

MIRI creates a pathological microenvironment characterized by danger signal release, excessive oxidative stress and ionic imbalance, which collectively serve as potent triggers for pyroptosis. Upon reperfusion, severe damage to cardiomyocytes causes the rapid release of abundant intracellular components such as ATP, mtDNA, high mobility group box 1 (HMGB1) and cellular debris [40], resulting in the activation of the NLRP3 inflammasome. Meanwhile, the sudden reintroduction of oxygen triggers a profound surge in ROS. This not only directly activates the NLRP3 inflammasome, but also damages mitochondria, leading to the release of mtDNA, which subsequently stimulates inflammasomes as a DAMP. Moreover, elevated ROS levels promote the production of inflammatory cytokines, thereby aggravating pyroptosis. Regarding ionic imbalance, K^+ ^efflux and Ca^2+^ overload occur during MIRI, and then induce the activation of NLRP3 and lead to pyroptosis. Recently, increasing evidence has proved that pyroptosis contributes significantly to MIRI and the increasing levels of inflammatory cytokines and many pyroptosis-related proteins during MIRI. Studies have shown that the levels of NLRP3, cleaved caspase-1, ASC, and GSDMD-N were significantly elevated in the hypoxia/reperfusion model. Moreover, inflammatory cytokines including IL-18 and IL-1β were also up-regulated [40,41].

Studies have found that hypoxia can induce HMGB1, a critical mediator of pyroptosis, while inhibiting the activation of nuclear factor erythroid 2-related factor 2 (Nrf2)/HO-1 pathway, thereby accelerating pyroptosis induced by NLRP3 and caspase-1, and exacerbating MIRI in cardiomyocytes [40]. In addition, Zhou et al. [42] demonstrated that in a rat model, pyroptosis and inflammation were associated with increased ROS production in cardiomyocytes. Moreover, accumulating evidence indicates that ionic disturbances, particularly calcium overload, play an essential role in pyroptosis. Zhu et al. [43] used high-dose remifentanil to treat the rats and cells, and found that the expression of calcium-sensing receptor (CaSR) increased. Overexpression of CaSR elevates intracellular calcium and ROS levels, thereby accelerating pyroptosis in cardiomyocytes. Similarly, Mo et al. [44] demonstrated that in the hypoxia/reperfusion model cells, the expression of inositol 1,4,5-trisphosphate receptor type 1 (IP3R1) is down-regulated, whereas endoplasmic reticulum protein 44 (ERP44) is overexpressed. The binding of IP3R1 to ERP44 can reduce calcium overload, inflammation and pyroptosis in cardiomyocytes. Furthermore, several non-classical pathways have recently been proposed. Studies have demonstrated that knockdown of HMGB1 could down-regulate the levels of inflammasomes and pyroptosis-related proteins; however, the overexpression of absent in melanoma 2 (AIM2) aggravates myocardial injury, inflammation and pyroptosis in endothelial cells [45]. Therefore, the HMGB1/AIM2 pathway plays an important role in pyroptosis.

Similar to their impact on ferroptosis, metabolic diseases also upregulate the susceptibility to pyroptosis in myocardial ischemia/reperfusion injury. Researchers have found that a high uric acid burden worsened the elevation of creatine kinase-MB (CK-MB), caspase-3 and pyroptosis-related proteins following myocardial ischemia/reperfusion. Mechanistically, uric acid activates the NLRP3 inflammasome and promotes the production of ROS, resulting in pyroptosis aggravation in cardiomyocytes [46].

## 5. The Crosstalk Between Ferroptosis and Pyroptosis in MIRI

**Fig. 1.**
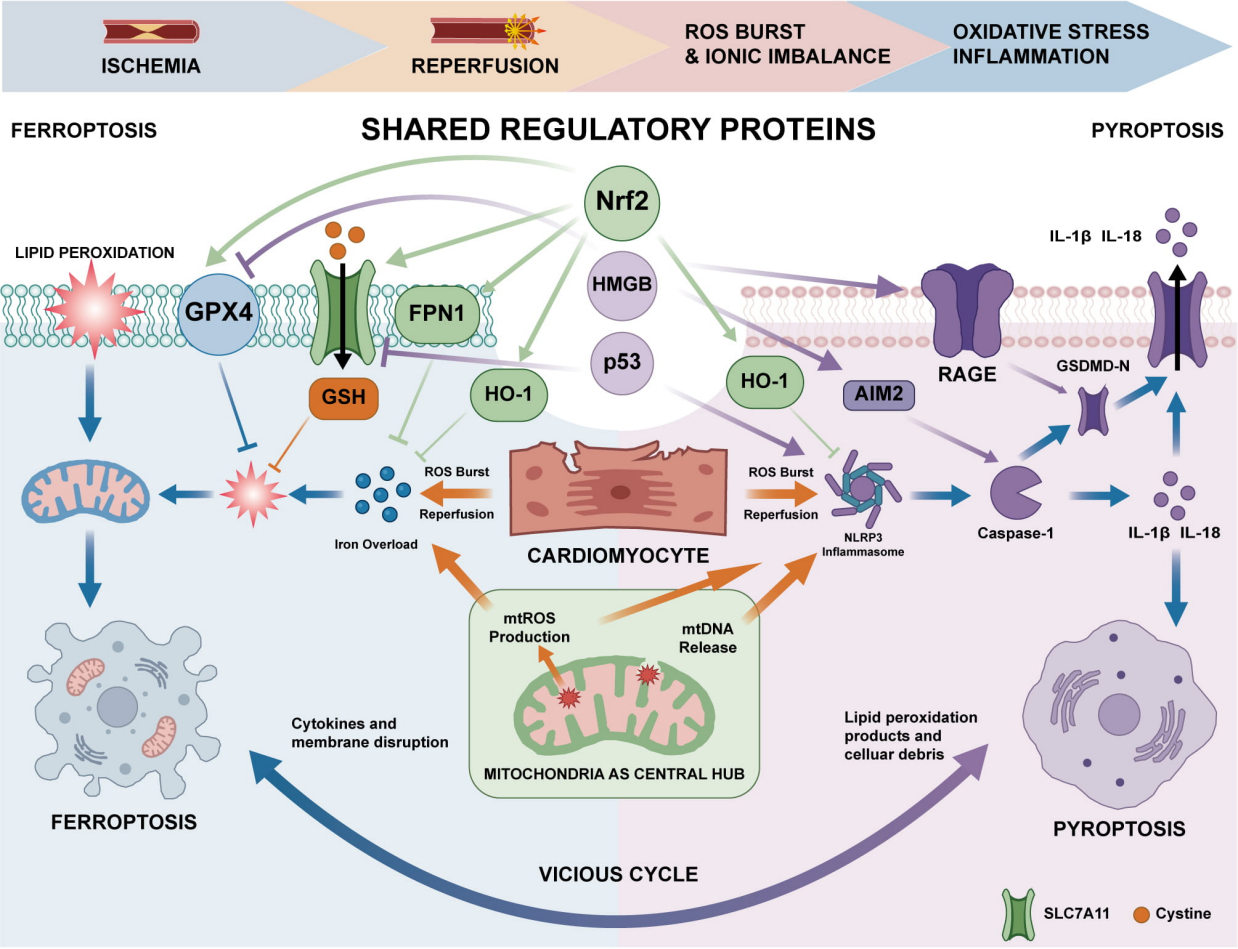
**The crosstalk between ferroptosis and pyroptosis in MIRI**. (Center) Shared regulatory proteins including Nrf2, p53, and HMGB act as molecular switches regulating both ferroptosis and pyroptosis. Nrf2 exerts a protective effect. Conversely, p53 and HMGB may promote cell death through distinct mechanisms. (Left) Ferroptosis is driven by iron overload and lipid peroxidation, leading to membrane rupture. (Right) Pyroptosis is mediated by the ROS/mtDNA-dependent activation of the NLRP3 inflammasome, leading to caspase-1 cleavage, GSDMD pore formation, and inflammatory cytokine release. (Bottom) Ferroptosis and pyroptosis may affect each other through interconnected inflammatory and oxidative feedback loops, thereby forming a vicious cycle. Abbreviation used: GSH, glutathione; FPN1, ferroportin 1; HO-1, heme oxygenase-1; Nrf2, nuclear factor erythroid 2–related factor 2; HMGB, high mobility group box; AIM2, absent in melanoma 2; RAGE, receptor for advanced glycation end products; GSDMD-N, gasdermin D N-terminal; IL-1β, interleukin 1 beta; IL-18, interleukin 18. This figure was generated using Adobe Illustrator.

To date, direct experimental evidence demonstrating a causal interaction between ferroptosis and pyroptosis in myocardial ischemia/reperfusion injury is scarce. Most available studies focus on either ferroptosis or pyroptosis independently, and only a limited number of reports in related cardiac conditions, such as heart failure [10] and myocardial fibrosis [47], suggest potential links between these pathways. Therefore, the proposed crosstalk in MIRI is primarily extrapolated from the co-activation of ferroptosis and pyroptosis under shared regulatory proteins and shared mechanisms, and mechanistic insights derived from non-cardiac conditions, such as drug resistance [11], epilepsy [9] and central nervous system injury [48]. To conceptualize these interconnected networks, we proposed a comprehensive model illustrating the mechanistic crosstalk between ferroptosis and pyroptosis in MIRI (Fig. 1).

### 5.1 Spatiotemporal Interplay Between Ferroptosis and Pyroptosis in MIRI

**Fig. 2.**
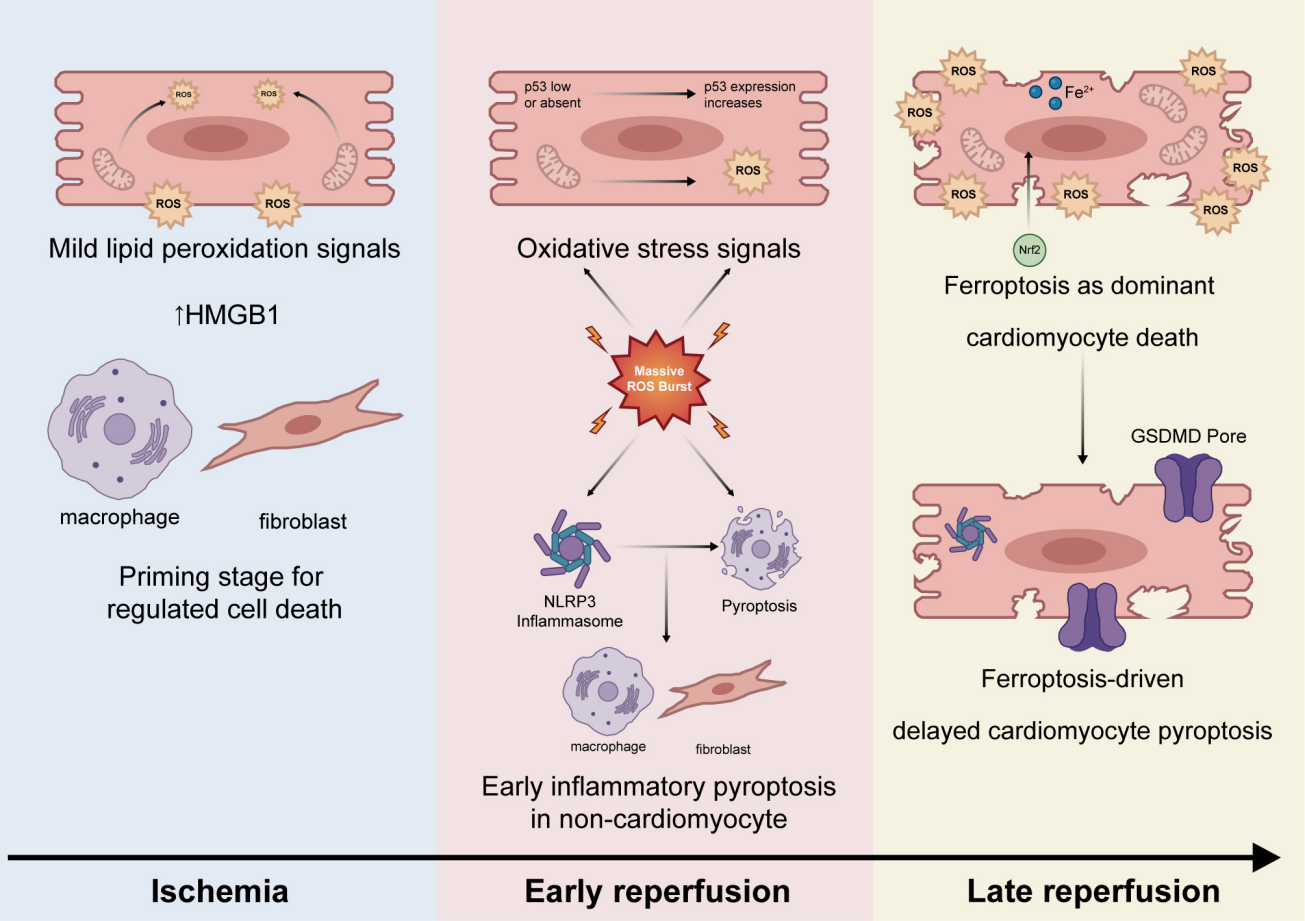
**Spatiotemporal interplay between ferroptosis and pyroptosis in MIRI**. (Left) Ischemia: The lack of oxygen restricts massive ROS production. This phase serves as a critical priming stage characterized by mild lipid peroxidation signals and the early up-regulation of HMGB1, without immediate cell rupture. (Middle) Early reperfusion: The sudden influx of oxygen induces a massive ROS burst. This acute oxidative stress initiates the assembly of the NLRP3 inflammasome, driving early inflammatory pyroptosis predominantly in non-cardiomyocytes, such as macrophages and fibroblasts. Concurrently, stress-sensing regulators like p53 transition from baseline to elevated expression levels. (Right) Late reperfusion: Synergistic intracellular iron overload and persistent ROS influx drive severe lipid peroxidation, rendering ferroptosis the dominant form of cardiomyocyte death. While compensatory Nrf2 activation occurs to counteract oxidative damage, the rupture of ferroptotic cells releases DAMPs into the microenvironment. This persistent danger signaling subsequently triggers a secondary, delayed wave of pyroptosis, within the cardiomyocytes. This figure was generated using Adobe Illustrator.

Accumulating evidence suggests that the shared regulators of ferroptosis and pyroptosis are activated in a phase-dependent manner during myocardial ischemia and reperfusion, rather than forming a single synchronized pathway. This temporal organization provides a useful framework to integrate how these two forms of regulated cell death may be linked during different stages of MIRI (Fig. 2).

During the ischemic phase, the lack of oxygen restricts massive ROS production, meaning that ferroptosis and pyroptosis do not fully execute. However, this phase serves as a critical priming stage. Xiang et al. [49] noted that ischemia initiates early lipid peroxidation signals without causing immediate cell rupture. At the same time, the expression of key regulatory proteins begins to change. For instance, the levels of HMGB1 increase substantially as early as 30 minutes after ischemia [50].

When reperfusion starts, the sudden influx of oxygen causes a massive ROS burst. In this acute phase, the initial wave of cell death is largely inflammatory. The ROS burst rapidly triggers the assembly of the NLRP3 inflammasome, which reaches a robust expression level within 1 hour of reperfusion. Notably, this early pyroptotic activation primarily occurs in non-cardiomyocytes, such as resident macrophages and fibroblasts, creating an initial pro-inflammatory microenvironment [51,52]. Concurrently, other stress-sensing regulators begin to accumulate. For example, the p53 expression remains at a very low level during the ischemic phase and the early period of reperfusion. However, its expression becomes detectable approximately 2 hours after reperfusion [53].

As reperfusion continues, the massive ROS influx, combined with intracellular iron overload, drives severe lipid peroxidation. Because cardiomyocytes are highly vulnerable to oxidative stress, they undergo rapid ferroptosis, which emerges as the dominant form of cardiomyocyte death during this phase [23,54]. As ferroptotic cardiomyocytes rupture, they release their cellular contents into the microenvironment. This includes HMGB1, which peaks at approximately 6 hours following reperfusion and remains elevated at 24 hours as well as several days later [50,55]. Meanwhile, delayed protective responses, such as the activation of Nrf2, are significantly induced around 6 hours post-reperfusion to counteract the ongoing oxidative damage [56]. The persistent release of ferroptosis-derived DAMPs, like HMGB1, acts as a continuous danger signal. These DAMPs exacerbate the inflammatory microenvironment, subsequently triggering a delayed, secondary wave of massive pyroptosis within the cardiomyocytes themselves.

### 5.2 Regulatory Proteins Shared by Ferroptosis and Pyroptosis

Recently, an increasing number of regulatory pathways and factors involved in ferroptosis and pyroptosis have been investigated. Notably, certain regulatory proteins contribute significantly not only to ferroptosis but also to pyroptosis. Here, we discuss several essential proteins regulating both ferroptosis and pyroptosis, as well as their potential relationships in MIRI.

#### 5.2.1 Nrf2

Nrf2 is a transcription factor that controls how cells fight against oxidative stress [57]. It regulates the expression of antioxidant genes, anti-inflammatory genes and metabolism re-programming genes. Under physiological conditions, kelch-like ECH-associated protein 1 (Keap1) sequesters Nrf2 in the cytoplasm and targets it for proteasomal degradation, thereby maintaining low levels of Nrf2 expression. However, when the cells are exposed to stress, Keap1 changes its shape and releases Nrf2. After that, Nrf2 moves into the nucleus and binds to the antioxidant response element (ARE), resulting in enhanced anti-oxidation and anti-inflammation [58].

During MIRI, the myocardium is under a strong stress condition, leading to up-regulated expression of Nrf2. Dong et al. [59] reported that the increased levels of Nrf2 can activate SLC7A11 and GPX4. SLC7A11 promotes the synthesis of GSH, while up-regulating GPX4 levels. Then, GPX4 plays an essential role in the anti-oxidative defense system with GSH, thereby inhibiting ferroptosis. Furthermore, studies have shown that increased Nrf2 expression is accompanied by elevated levels of ferroportin 1 (FPN1). Activation of the Nrf2/FPN1 pathway induces Fe^2+ ^efflux and reduces iron overload, thereby maintaining iron homeostasis [60]. Studies have also reported that up-regulating Nrf2 markedly increases HO-1 expression, which down-regulates ROS production and alleviates ferroptosis [61]. Nevertheless, excessive up-regulation of HO-1 can induce Fe^2+ ^accumulation and aggravate ferroptosis [62]. Furthermore, the role of G-protein signaling 12 (RGS12)/Nrf2 pathway is another critical pathway regulating ferroptosis in MIRI [63].

With regard to pyroptosis, the Nrf2/HO-1 pathway is also important. Researchers have found that the inhibition of Nrf2/HO-1 increases the expression of ASC, caspase-1 and NLRP3, all of which are essential regulators of pyroptosis. Additionally, the levels of IL-18 and IL-1β are up-regulated. Consequently, pyroptosis is accelerated, leading to exacerbating MIRI [40].

#### 5.2.2 HMGB

HMGB1 and high mobility group box 2 (HMGB2) both belong to the high mobility group (HMG) protein family. Emerging evidence has reported that both HMGB1 and HMGB2 are both significant regulators in MIRI [40,45].

Under physiological conditions, HMGB1 is responsible for maintaining chromosomal stability and facilitating DNA repair. In contrast, under pathological conditions, HMGB1 acts as a critical DAMP [64]. Zhu et al. [65] found that HMGB1 is markedly elevated in ischemia/reperfusion models, where it interacts with toll-like receptor 4 (TLR4) to increase the production of inflammatory cytokines. Researchers have also proved that the inhibition of HMGB1 up-regulates GPX4 expression and down-regulates ferroptosis-related proteins expression, thereby reducing ferroptosis [65]. Moreover, studies have reported that HMGB1 knockdown leads to the suppression of AIM2-related inflammation and pyroptosis-related protein expression [45]. Additionally, the HMGB1/NLRP3/caspase-1 pathway and the HMGB1/receptor for advanced glycation end products (RAGE)/GSDMD pathway are closely associated with pyroptosis in MIRI [40,66].

The functions of HMGB2 include chromatin remodeling and the regulation of gene expression [67]. Studies have suggested that the expression of HMGB2 increased markedly in the hypoxia/reperfusion cell model. Kruppel-like factor 9 (KLF9) bound to the promoter region of HMGB2 and enhanced the transcription of HMGB2, subsequently up-regulating its expression and leading to ferroptosis and inflammation [68].

Taken together, members of the HMGB protein family act as vital molecular switches that control both ferroptosis and pyroptosis. Its mechanisms and functions in MIRI deserve further research and investigation.

#### 5.2.3 p53

p53 is a classical stress-sensing transcription factor. During MIRI, p53 can be robustly activated during both the ischemic phase (hypoxia) and the reperfusion phase (characterized by a burst of ROS and ionic disturbances) [69]. Focusing on ferroptosis, the most classical regulatory axis is the p53/SLC7A11 pathway. p53 suppresses the expression of SLC7A11, thereby decreasing GSH synthesis and GPX4 activity. Subsequently, the disruption of the GSH/GPX4 antioxidant system exacerbates ferroptosis [70]. Recently, Gong et al. [71] demonstrated that ubiquitin-specific peptidase 38 (USP38) is up-regulated in a mouse model of I/R injury, modulating the p53/SLC7A11 pathway to aggravate ferroptosis. However, not all p53 activation promotes ferroptosis. A previous study has reported that p53 can inhibit dipeptidyl peptidase 4 (DPP4)-NADPH oxidase 1 (NOX1) complex-mediated lipid peroxidation, thereby reducing ferroptosis [72]. In pyroptosis, studies have demonstrated that the activation of p53 decreased autophagosome clearance by regulating the p53/BCL2/adenovirus E1B 19 kDa protein-interacting protein 3 (BNIP3) pathway, and down-regulated the degradation of NLRP3, ultimately leading to pyroptosis [73]. Therefore, p53 may represent an important therapeutic target in MIRI through its dual regulation of both ferroptosis and pyroptosis.

### 5.3 Potential Relationship Between Ferroptosis and Pyroptosis

Although most researchers investigated the mechanisms of ferroptosis and pyroptosis in MIRI independently, emerging evidence indicates that ferroptosis and pyroptosis may share regulatory pathways. Notably, ROS acts as a critical upstream trigger for both ferroptosis and pyroptosis, while mitochondrial dysfunction serves as a central hub integrating oxidative stress, metabolic disturbances, and inflammatory signaling. The following sections discuss the potential mechanistic links between ferroptosis and pyroptosis in MIRI.

#### 5.3.1 ROS as a Shared Upstream Trigger of Ferroptosis and Pyroptosis

The excessive production of ROS represents one of the earliest and most critical pathological events in MIRI, and serves as a common upstream trigger linking ferroptosis and pyroptosis. During the early ischemic phase, ROS generation is initiated, and subsequently undergoes a pronounced burst at the onset of reperfusion [74]. In the context of ferroptosis, excessive ROS drives iron-dependent lipid peroxidation, resulting in oxidative damage to cellular and mitochondrial membranes. When endogenous antioxidant defense systems, particularly the GPX4/GSH axis, are overwhelmed or impaired, lipid peroxidation accumulates and renders cardiomyocytes highly susceptible to ferroptosis. Beyond ferroptosis, ROS also function as key signaling molecules that promote pyroptotic activation. Elevated ROS levels facilitate the activation of the NLRP3 inflammasome, either directly or indirectly through ROS-induced mitochondrial dysfunction and the release of mitochondrial danger signals. Subsequent activation of the NLRP3/caspase-1 pathway leads to GSDMD-mediated membrane pore formation and inflammatory cell death. Importantly, ROS may not only act as parallel triggers for ferroptosis and pyroptosis, but also serve as a central signaling hub that integrates oxidative damage and inflammatory responses during MIRI. Therefore, ROS-driven signaling serves as the fundamental link connecting these two cell death pathways during reperfusion.

#### 5.3.2 Mitochondrial Dysfunction as a Central Hub Linking Ferroptosis and Pyroptosis

As a highly energy-demanding organ, the heart relies heavily on mitochondrial oxidative metabolism, resulting in a high mitochondrial density in cardiomyocytes. Increasing evidence reports that mitochondria serve as a critical hub linking ferroptosis and pyroptosis during MIRI. During MIRI, mitochondrial dysfunction leads to the excessive production of mitochondrial reactive oxygen species (mtROS), particularly upon reperfusion [75]. Elevated mtROS promotes iron-dependent lipid peroxidation and contributes to mitochondrial membrane damage, thereby sensitizing cardiomyocytes to ferroptotic cell death. Meanwhile, mtROS act as potent signaling molecules that facilitate the activation of the NLRP3 inflammasome, which subsequently triggers caspase-1 activation and pyroptotic cell death. In addition to mtROS, mitochondrial damage induced by lipid peroxidation leads to the translocation of mtDNA into the cytosolic and extracellular spaces. Acting as a DAMP, mtDNA further aggravates inflammatory responses and exacerbates pyroptosis by promoting inflammasome activation. Collectively, mitochondrial dysfunction integrates oxidative stress, iron metabolism, and inflammatory signaling, thereby establishing mitochondria as a central platform for the crosstalk between ferroptosis and pyroptosis during MIRI.

#### 5.3.3 Oxidative-Inflammatory Feedback Loops between Ferroptosis and Pyroptosis

Rather than occurring as independent events, ferroptosis and pyroptosis may affect each other through interconnected inflammatory and oxidative feedback loops. Lipid peroxidation products and cellular debris generated during ferroptosis can function as danger signals, thereby enhancing inflammatory responses and facilitating pyroptotic activation. Conversely, pyroptosis-associated cytokine release and membrane disruption aggravate oxidative stress and disturb Fe^2+^ homeostasis, which increases susceptibility to ferroptosis. Together, these processes form a vicious cycle that contributes to persistent cardiomyocyte injury and inflammation during MIRI.

#### 5.3.4 Cell Type-Specific and Intercellular Mechanisms Linking Ferroptosis and Pyroptosis in MIRI

**Fig. 3.**
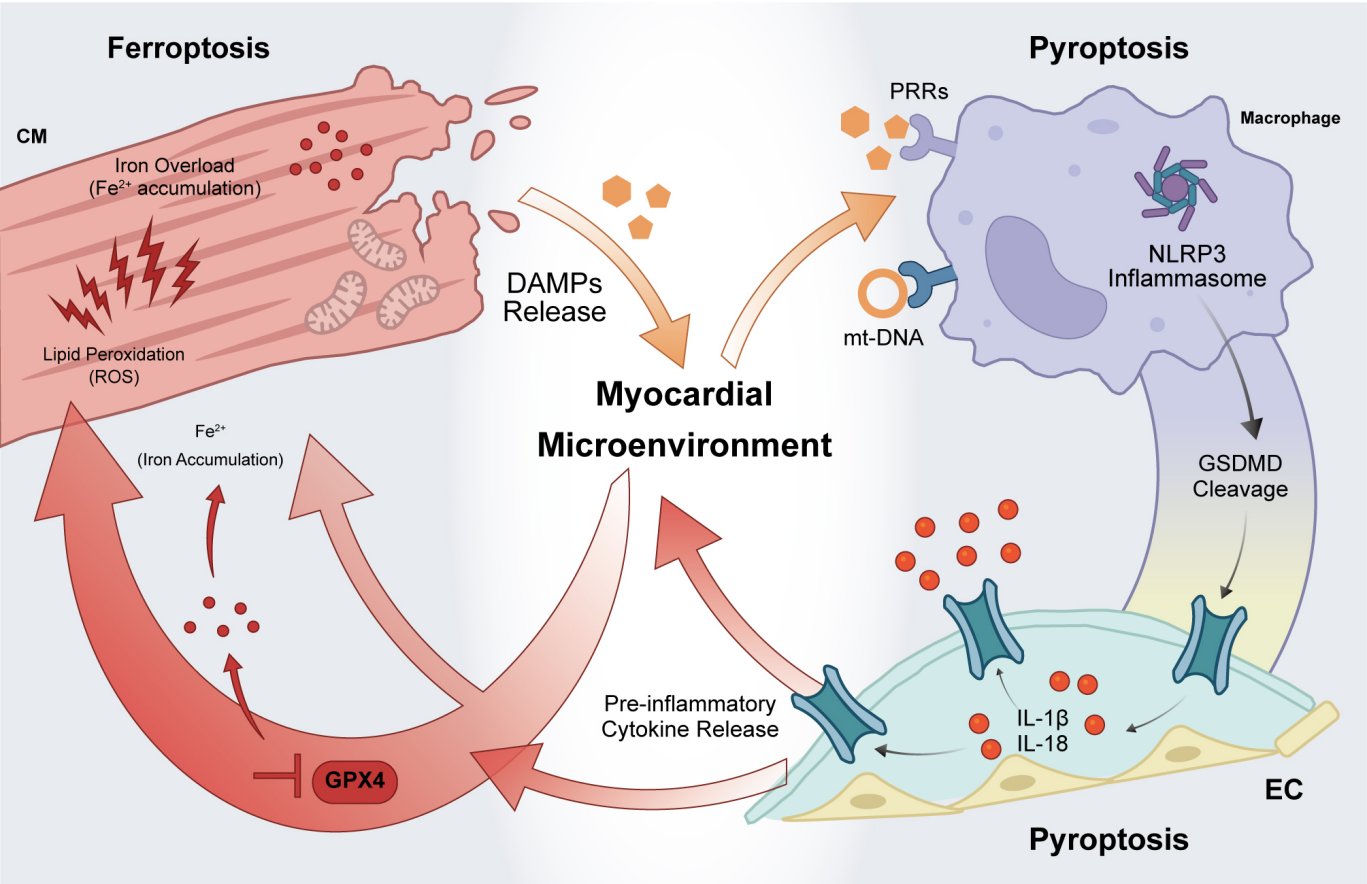
**Intercellular crosstalk between ferroptosis and pyroptosis in MIRI**. Cardiomyocytes (CM) predominantly undergo ferroptosis driven by iron overload and lipid peroxidation, subsequently releasing DAMPs and mitochondrial DNA (mt-DNA) into the myocardial microenvironment. These danger signals are recognized by pattern recognition receptors (PRRs) on neighboring macrophages, triggering NLRP3 inflammasome activation and GSDMD cleavage. This cascade leads to pyroptosis in macrophages and endothelial cells (EC), accompanied by the massive release of pro-inflammatory cytokines, such as IL-1β and IL-18. These cytokines establish a vicious positive feedback loop by suppressing the anti-oxidative system and promoting further iron accumulation in surviving CMs, thereby sensitizing them to subsequent ferroptosis and amplifying myocardial injury. This figure was generated using Adobe Illustrator.

Myocardial ischemia/reperfusion injury is a multicellular pathological process involving cardiomyocytes, endothelial cells, immune cells, and cardiac fibroblasts [49]. Importantly, the execution of ferroptosis and pyroptosis exhibits distinct cellular heterogeneity within the myocardial microenvironment. Accumulating evidence indicates that ferroptosis predominantly affects cardiomyocytes in MIRI [76], where excessive lipid peroxidation and iron-dependent oxidative damage compromise membrane integrity. In contrast, pyroptosis is not only observed in cardiomyocytes but also in non-cardiomyocytes, such as macrophages and endothelial cells [45,77]. This cell-type-specific distribution suggests that ferroptosis and pyroptosis may be functionally linked through intercellular communication (Fig. 3).

Ferroptotic cardiomyocytes can release a range of DAMPs, including HMGB1 and mitochondrial DNA, into the myocardial microenvironment [78]. These DAMPs are recognized by pattern recognition receptors expressed by neighboring macrophages and endothelial cells, thereby promoting NLRP3 inflammasome activation and subsequent pyroptotic signaling [34]. Through this mechanism, ferroptotic injury in cardiomyocytes may indirectly amplify inflammatory cell death and inflammatory responses in non-myocyte populations.

Conversely, the pyroptosis of macrophages significantly exacerbates the ferroptotic sensitivity of surrounding cardiomyocytes and endothelial cells. Upon GSDMD-dependent membrane pore formation, pyroptotic macrophages secrete massive quantities of pro-inflammatory cytokines, notably IL-1β and IL-18. In the MIRI microenvironment, IL-1β and IL-18 may contribute to intracellular iron dysregulation, oxidative stress, and impairment of ferroptosis defense systems, including GPX4-dependent antioxidant capacity, in neighboring cardiomyocytes and microvascular endothelial cells [79]. This macrophage-driven inflammatory microenvironment establishes a vicious cycle, sensitizing surviving cardiomyocytes and endothelial cells to subsequent ferroptosis.

## 6. Therapeutic Strategies Targeting Ferroptosis and Pyroptosis in MIRI

### 6.1 Therapeutic Strategies Targeting Ferroptosis

Therapeutic strategies targeting ferroptosis in MIRI have mainly centered on regulating iron metabolism, inhibiting lipid peroxidation and enhancing the antioxidant defense system. Therapeutic strategies can be broadly categorized into pharmacological and non-pharmacological interventions.

Deferasirox, a ferric iron chelator, has been reported to suppress ferroptosis by regulating iron metabolism in cardiomyocytes [80]. Specifically, it downregulates the levels of iron in the endoplasmic reticulum, thereby decreasing myocardial infarct size. Moreover, combination therapy with cyclosporin A markedly inhibits ferroptosis and MIRI, with particular efficacy in attenuating adverse cardiac remodeling after ischemia/reperfusion injury. Zhu et al. [81] demonstrated that piceatannol, an active compound derived from traditional Chinese medicine, effectively alleviates cardiomyocyte ferroptosis in MIRI through the Nrf2/FPN1/transferrin receptor 1 (TfR-1) pathway. Upon the activation of Nrf2, FPN1 expression is significantly up-regulated, whereas TfR-1 expression is down-regulated after piceatannol treatment, leading to a reduction in iron overload and subsequent amelioration of MIRI. In addition, nobiletin [82] have been shown to alleviate ferroptosis in MIRI via the ACSL4/NCOA4 pathway in cardiomyocytes.

Previous studies have found that delphinidin inhibits ferroptosis in MIRI through down-regulating the expression of ALOX15, an enzyme that promotes the accumulation of phospholipid hydroperoxides and thereby exacerbates ferroptotic cell death in cardiomyocytes [20,83]. Furthermore, a non-pharmacological strategy known as mild therapeutic hypothermia protection has been found to reduce cardiomyocyte ferroptosis in MIRI by decreasing ACSL4 and transient receptor potential melastatin 7 (TRPM7) expression via the phosphoinositide 3-kinase (PI3K)/protein kinase B (AKT) pathway, both of which are closely related to lipid peroxidation [84].

Emerging evidence suggests that the enhancement of the antioxidant defense system represents a critical treatment for ferroptosis in MIRI. Melatonin, best known for regulating circadian rhythms, has shown significant promise in ferroptosis treatment. He et al. [85] demonstrated that melatonin down-regulates activating transcription factor 3 (ATF3) expression, thereby promoting GPX4 expression in cardiomyocytes. Meanwhile, Wang et al. [21] have shown that melatonin inhibits the activation of the ATM/p53 pathway, and increases the expression of downstream antioxidant-related proteins, including GPX4, superoxide dismutase 2 (SOD-2), cystine/glutamate antiporter (XCT) and FTH1, resulting in enhancing antioxidant defense system and alleviating ferroptosis and MIRI in cardiomyocytes. Dexmedetomidine, a specific agonist for α_2_-adrenergic receptors, has also been considered a potential therapeutic agent for ferroptosis. Accumulating evidence indicates that dexmedetomidine attenuates cardiomyocyte ferroptosis in MIRI by enhancing antioxidant capacity through the modulation of multiple signaling pathways, including Nrf2/SLC7A11/GPX4 [86,87], and Akt/mechanistic target of rapamycin (mTOR)/Nrf2 axes [88]. Moreover, several non-pharmacological therapeutic strategies have been reported, including acupuncture [89] and vagus nerve stimulation [90]. Acupuncture alleviates ferroptosis via the Nrf2/HO-1 signaling pathway in cardiomyocytes, whereas vagus nerve stimulation mitigates ferroptosis by activating the SLC7A11/GPX4 pathway in cardiomyocytes.

### 6.2 Therapeutic Strategies Targeting Pyroptosis

Therapeutic strategies targeting pyroptosis in MIRI can be classified into those inhibiting either the classical or non-classical pyroptotic pathways. Similar to ferroptosis treatments, these strategies can also be broadly categorized into pharmacological and non-pharmacological approaches.

Pharmacological inhibition of the NLRP3 inflammasome represents one of the most extensively investigated therapeutic approaches. Dexmedetomidine has been shown not only to reduce ferroptosis in MIRI but also to suppress pyroptosis. Specifically, it inhibits cardiomyocyte pyroptosis by regulating NLRP3 expression via the miR-665/myocyte enhancer factor 2D (MEF2D)/Nrf2 pathway [91]. Trehalose, a natural non-reducing disaccharide, has also been reported to alleviate MIRI in cardiomyocytes by reducing NLRP3-mediated pyroptosis in cardiomyocytes [92]. In addition, growing evidence indicates that several traditional Chinese medicines exert protective effects against pyroptosis in MIRI. These include rutin, which modulates the nuclear factor kappa B (NF-κB)/NLRP3 pathway in cardiomyocytes [93]; aesculin, which acts through the Akt/glycogen synthase kinase 3 beta (GSK3β)/NF-κB pathway in cardiomyocytes [94]; and chlorogenic acid, which suppresses pyroptosis through the long non-coding RNA nuclear paraspeckle assembly transcript 1 (LncRNA NEAT1)/NLRP3 axis in cardiomyocytes [95]. Beyond NLRP3 inflammasome inhibition, the direct targeting of gasdermins (GSDMs), the key executors of pyroptotic cell death, has gained increasing attention. Lin et al. [96] reported that oridonin down-regulates the levels of pyroptosis-mediated proteins like NLRP3 and ASC, resulting in reducing GSDMD-mediated pyroptosis in MIRI in cardiomyocytes. Trimetazidine, a widely used anti-ischemia drug for coronary heart disease, has been found to suppress GSDMD-mediated pyroptosis in MIRI by regulating the TLR4/myeloid differentiation primary response 88 (MyD88)/NF-κB/NLRP3 pathway in cardiomyocytes [97]. In addition to pharmacological interventions, several non-pharmacological strategies targeting the classical pathway contribute to inhibiting pyroptosis in MIRI. Li et al. [98] reported that aerobic exercise down-regulates the level of insulin-like growth factor binding protein 2 (IGFBP2), thereby alleviating GSDME-mediated pyroptosis via the Akt/GSK3β pathway in cardiomyocytes. Moreover, researchers found that magnetic vagus nerve stimulation plays an essential role in suppressing pyroptosis via the muscarinic acetylcholine receptor M2 (M2AChR)/oxoglutarate dehydrogenase-like (OGDHL)/ROS axis in cardiomyocytes [99].

Focusing on the non-classical pathway, Wang et al. [66] demonstrated that non-anticoagulant heparin binds to HMGB1, thereby blocking the HMGB1/RAGE pathway in cardiomyocytes. This interaction results in decreased activation of GSDMD and caspase-11, ultimately ameliorating pyroptosis. Furthermore, gemfibrozil, a drug used for treating hyperlipidemia, has been reported to mitigate cardiomyocyte pyroptosis by down-regulating the levels of GSDMD-N and caspase-11 [100].

### 6.3 Dual-Targeting Strategies Against Ferroptosis and Pyroptosis

Currently, therapeutic strategies that directly target both ferroptosis and pyroptosis in MIRI remain limited. Most existing studies have focused on interventions aimed at either ferroptotic or pyroptotic pathways individually. However, accumulating evidence indicates that ferroptosis and pyroptosis share several upstream regulatory mechanisms, as we discussed previously, including common signaling proteins, excessive ROS production, and mitochondrial dysfunction. These shared pathways provide a strong mechanistic basis for the development of dual-targeting therapies. To date, only a limited number of studies have directly demonstrated pharmacological agents capable of simultaneously modulating both ferroptosis and pyroptosis. For example, stachyose, an oligosaccharide found in traditional Chinese medicinal herbs, has been reported to decrease the levels of iron and increase GPX4 expression, thereby enhancing the antioxidant defense system and inhibiting ferroptosis in MIRI in cardiomyocytes. Moreover, it can also alleviate pyroptosis by down-regulating the levels of NLRP3, caspase-1, GSDMD, IL-18, and IL-1β in macrophages [77].

Based on the modulation of shared regulatory proteins such as Nrf2, p53, and HMGB, agents targeting these molecules may represent promising strategies for dual targeting. As discussed above, Nrf2 functions as an important regulatory protein in ferroptosis and pyroptosis by modulating antioxidant defenses and inflammatory responses. Leng et al. [101] reported that the main active components of *Prunella vulgaris L.* exert effects against MIRI by activating the Nrf2/GPX4 pathway, resulting in suppressing oxidative stress and ferroptosis. Given the central role of Nrf2 as a shared regulator of ferroptosis and pyroptosis, activation of Nrf2 by *Prunella vulgaris L.* may not only suppress ferroptosis but also potentially attenuate pyroptosis by limiting ROS accumulation and downstream inflammasome activation. Similarly, agents targeting HMGB1, such as glycyrrhizin [65] and non-anticoagulant heparin [66] may also play an essential role in treating both ferroptosis and pyroptosis. Moreover, therapeutic interventions aimed at reducing excessive ROS production and preserving mitochondrial function are likely to modulate ferroptotic and pyroptotic pathways. Representative examples include naringin [102], which improves mitochondrial dysfunction and *Lactobacillus* [103], which down-regulates the levels of ROS.

Notably, several interventions discussed separately in the context of ferroptosis or pyroptosis may exert dual regulatory effects. Dexmedetomidine and vagus nerve stimulation have both been reported to attenuate ferroptotic and pyroptotic signaling in myocardial ischemia/reperfusion injury. Although these interventions were often investigated from distinct mechanistic perspectives, their ability to reduce oxidative stress and suppress inflammatory responses suggests a common mechanistic basis for dual targeting of ferroptosis and pyroptosis.

To better summarize current therapeutic strategies, Table 2 (Ref. [21,66,77,80,81,82,83,84,85,86,88,89,90,91,92,93,94,95,96,97,98,99,100]) provides an overview of representative interventions targeting ferroptosis, pyroptosis, or both in myocardial ischemia/reperfusion injury. Building on our previous discussion of the temporal stages, interventions targeting inflammation and pyroptosis may have the best therapeutic window during the early phase of reperfusion. As reperfusion continues into the later phases, dual-targeting strategies against both ferroptosis and pyroptosis could be more effective.

**Table 2.** **Therapeutic strategies targeting ferroptosis and pyroptosis in MIRI**.

Intervention	Regulated cell death	Primary target/pathway	Model	Evidence level	Reference
Deferasirox	Ferroptosis	/	mice/NRVMs	*in vitro* + *in vivo*	[80]
Piceatannol	Ferroptosis	Nrf2/FPN1/TfR-1	mice/AC16	*in vitro* + *in vivo*	[81]
					
Nobiletin	Ferroptosis	ACSL4/GPX4/NCOA4	rats/H9c2	*in vitro* + *in vivo*	[82]
Delphinidin	Ferroptosis	ALOX15	rats/H9c2	*in vitro* + *in vivo*	[83]
Mild therapeutic hypothermic protection	Ferroptosis	PI3K/AKT	rats/H9c2	*in vitro* + *in vivo*	[84]
Melatonin	Ferroptosis	ATF3/GPX4 ATM/p53	rats/H9c2/HL-1	*in vitro* + *in vivo*	[21,85]
Acupuncture	Ferroptosis	Nrf2/HO-1	mice/H9c2	*in vitro* + *in vivo*	[89]
Trehalose	Pyroptosis	NLRP3	mice/NMVCs	*in vitro* + *in vivo*	[92]
Rutin	Pyroptosis	NF-κB/NLRP3	mice/primary mouse CMs	*in vitro* + *in vivo*	[93]
Aesculin	Pyroptosis	Akt/GSK3β/NF-κB	rats/NRCMs	*in vitro* + *in vivo*	[94]
Chlorogenic acid	Pyroptosis	Lnc Neat1/NLRP3	mice/HL-1	*in vitro* + *in vivo*	[95]
Oridonin	Pyroptosis	NF-κB/NLRP3	mice/NRCMs	*in vitro* + *in vivo*	[96]
Trimetazidine	Pyroptosis	TLR4/MyD88/NF-κB/NLRP3	rats/H9c2	*in vitro* + *in vivo*	[97]
Aerobic exercise	Pyroptosis	AKT/GSK3β	mice/primary mouse CMs	*in vitro* + *in vivo*	[98]
Non-anticoagulant heparin	Pyroptosis	HMGB1/RAGE	mice/H9c2	*in vitro* + *in vivo*	[66]
Gemfibrozil	Pyroptosis	caspase-11, GSDMD-N	mice/primary adult mouse CMs	*in vitro* + *in vivo*	[100]
Stachyose	Ferroptosis and Pyroptosis	/	mice/H9c2/RAW264.7	*in vitro* + *in vivo*	[77]
Dexmedetomidine	Ferroptosis and Pyroptosis	Nrf2/SLC7A11/GPX4 Akt/mTOR/Nrf2 miR-665/MEF2D/Nrf2	rats/H9c2	*in vivo*/*in vitro* + *in vivo*/*in vitro* + *ex vivo* + *in vivo*	[86,88,91]
Vagus nerve stimulation	Ferroptosis and Pyroptosis	SLC7A11/GPX4 M2AChR/OGDHL/ROS	rats/NRCMs	*in vivo*/*in vitro* + *in vivo*	[90,99]

Abbreviation used: TfR-1, transferrin receptor 1; NCOA4, nuclear receptor coactivator 4; PI3K, phosphoinositide 3-kinase; AKT, protein kinase B; ATF3, activating transcription factor 3; ATM, ataxia telangiectasia mutated; NF-κB, nuclear factor kappa B; GSK3β, glycogen synthase kinase 3 beta; Lnc Neat1, long non-coding RNA nuclear paraspeckle assembly transcript 1; TLR4, toll-like receptor 4; MyD88, myeloid differentiation primary response 88; mTOR, mechanistic target of rapamycin; MEF2D, myocyte enhancer factor 2D; M2AChR, muscarinic acetylcholine receptor M2; OGDHL, oxoglutarate dehydrogenase-like; NRVMs, neonatal rat ventricular myocytes; AC16, AC16 human cardiomyocyte cell line; HL-1, HL-1 cardiomyocyte cell line; NMVCs, neonatal mouse ventricular cardiomyocytes; H9c2, H9c2(2-1) rat cardiomyoblast cell line.

### 6.4 Limitations and Challenges in Clinical Translation

Despite promising preclinical findings, a substantial gap remains between experimental success and clinical translation of therapeutic strategies targeting ferroptosis and pyroptosis in MIRI.

A major limitation of current therapeutic evidence arises from the heavy reliance on standardized experimental models with generally good health status and limited comorbidities. Most studies investigating interventions targeting ferroptosis and pyroptosis in MIRI are based on rodent models or cardiomyocyte cell lines, such as H9c2 or HL-1 cells. However, significant interspecies differences exist between rodents and humans. For example, mice exhibit substantially higher heart rates and metabolic demands, as well as distinct antioxidant capacities and responses to MIRI and responsiveness to therapeutic interventions. Importantly, data from large animal models, such as porcine or non-human primate models, are largely lacking. Moreover, clinical trial evidence targeting ferroptosis and pyroptosis in MIRI is currently absent.

Another critical barrier to clinical translation is the difference between experimental treatment paradigms and clinical feasibility. Many pharmacological and non-pharmacological interventions, including dexmedetomidine, melatonin and acupuncture, demonstrate maximal efficacy when administered as preconditioning strategies prior to ischemic onset. In contrast, patients with acute myocardial infarction typically present after ischemia has already occurred, limiting therapeutic opportunities to the reperfusion phase. Consequently, interventions that rely on pre-ischemic administration are of limited clinical relevance. Furthermore, many natural compounds that exhibit anti-ferroptotic or anti-pyroptotic effects in experimental models suffer from poor aqueous solubility, rapid metabolism, and low oral bioavailability. Achieving therapeutically effective concentrations in cardiac tissue without inducing systemic toxicity remains a major challenge.

Although dual-targeting strategies that simultaneously modulate ferroptosis and pyroptosis are appealing, their clinical translation remains challenging. As discussed previously, ferroptosis and pyroptosis not only differ in their activation timing, but also preferentially occur in distinct cell populations. Ferroptosis predominantly affects cardiomyocytes at the late reperfusion phase, whereas pyroptosis is initially activated in non-cardiomyocytes, such as resident macrophages and fibroblasts during early reperfusion. Therefore, this combination of temporal mismatch and cellular heterogeneity significantly complicates the identification of an optimal therapeutic window for simultaneous interventions. Moreover, simultaneous modulation of oxidative stress, iron metabolism, and inflammatory signaling increases concerns regarding dosing complexity and off-target effects. For example, systemic inhibition of ferroptosis through iron chelation, like deferasirox, while effective in reducing lipid peroxidation, may disrupt physiological iron homeostasis and impair essential cellular functions, leading to anemia or immune dysfunction. Similarly, excessive suppression of pyroptosis and inflammasome signaling may interfere with essential inflammatory and reparative processes following myocardial infarction.

## 7. Limitations

Several limitations should be acknowledged in the current research as well as in this review.

Many studies investigating ferroptosis or pyroptosis in MIRI focus on a single form of cell death in isolation. Direct experimental evidence simultaneously examining the causal relationship between ferroptosis and pyroptosis in the same cardiac model remains scarce. Moreover, the temporal and cell type-specific dynamics of ferroptosis and pyroptosis during MIRI are still incompletely characterized. Most studies assess molecular markers at limited time points, making it difficult to precisely define the sequence, duration, and relative contribution of each form of cell death across different phases of ischemia and reperfusion. In addition, the heterogeneous involvement of cardiomyocytes, immune cells, endothelial cells, and fibroblasts further complicates the interpretation of experimental results. Furthermore, most existing evidence regarding ferroptosis and pyroptosis in MIRI is derived from *in vitro* experiments or small animal models, predominantly rodents. The lack of data from large-animal models and well-designed clinical trials limits the direct translational relevance of current findings.

This article itself has certain methodological limitations. This review was conducted as a narrative review rather than a systematic review or meta-analysis, meaning the literature selection may be subject to inherent selection bias. Additionally, our literature search was restricted to articles published in English, which may exclude relevant and significant findings published in other languages. Because the investigation of regulated cell death is a rapidly and continuously evolving field, some very recent discoveries or unpublished data might not be captured in the present study.

## 8. Conclusion and Future Perspectives

MIRI remains a major clinical challenge and is characterized by complex and multifactorial mechanisms of cardiomyocyte injury. Recent studies have demonstrated that regulated cell death plays a central role in MIRI. Among these, ferroptosis and pyroptosis have emerged as two important forms of cell death that contribute to cardiomyocyte and inflammatory injury during ischemia and subsequent reperfusion. These two forms of cell death are closely interconnected through shared upstream pathological mechanisms, including excessive ROS generation, mitochondrial dysfunction, and common regulatory proteins such as Nrf2, p53, and HMGB family members. From a therapeutic perspective, recognition of the interplay between ferroptosis and pyroptosis offers important implications for the development of more effective cardioprotective strategies.

Future studies should move beyond examining ferroptosis and pyroptosis as isolated events and instead focus on their dynamic and coordinated regulation during ischemia and reperfusion. Clarifying the temporal sequence, causal relationship, and cell type-specific involvement of these two forms of regulated cell death remains a key research priority. Importantly, increasing attention should be directed toward key shared regulators. Among these, Nrf2, HMGB1 and p53 emerge as particularly promising candidates, as they participate in both ferroptosis and pyroptosis and may function as molecular switches during different stages of MIRI. Targeting such shared regulators may offer a rational strategy for simultaneously modulating multiple forms of cell death. From a translational perspective, future therapeutic strategies should emphasize clinically relevant intervention windows, particularly during the reperfusion phase. Approaches modulating shared upstream regulators, rather than single downstream pathways, may provide more effective and durable cardioprotection.

## Abbreviations

IHD, ischemic heart disease; AMI, acute myocardial infarction; PCI, percutaneous coronary intervention; MIRI, myocardial ischemia/reperfusion injury; COPD, chronic obstructive pulmonary disease; ROS, reactive oxygen species; PUFAs, polyunsaturated fatty acids; ER, endoplasmic reticulum; GSH, glutathione; GPX4, glutathione peroxidase 4; SOX4, SRY-box transcription factor 4; DAPK, death-associated protein kinase; ALOX15, arachidonate 15-lipoxygenase; 15-HpETE, 15-hydroperoxyeicosatetraenoic acid; ATM, ataxia telangiectasia mutated; HO-1, heme oxygenase-1; ZIP14, Zrt- and Irt-like protein 14; PGC-1α, peroxisome proliferator-activated receptor gamma coactivator 1 alpha; RNF34, ubiquitin ligase ring finger protein 34; ACSL4, acyl-CoA synthetase long-chain family member 4; dsDNA, double-stranded DNA; cGAS, cyclic GMP-AMP synthase; STING, stimulator of interferon genes; SLC7A11, solute carrier family 7 member 11; NCOA4, nuclear receptor coactivator 4; FTH1, ferritin heavy chain 1; Rev-erbα, nuclear receptor subfamily 1 group D member 1; DAMPs, damage-associated molecular patterns; PAMPs, pathogen-associated molecular patterns; ATP, adenosine triphosphate; mtDNA, mitochondrial DNA; LPS, lipopolysaccharide; NLRP3, NOD-like receptor family pyrin domain containing 3; ASC, apoptosis-associated speck-like protein containing a CARD; GSDMD, gasdermin D; IL-1β, interleukin 1 beta; IL-18, interleukin 18; GSDMD-N, gasdermin D N-terminal; HMGB1, high mobility group box 1; Nrf2, nuclear factor erythroid 2–related factor 2; CaSR, calcium-sensing receptor; IP3R1, inositol 1,4,5-trisphosphate receptor type 1; ERP44, endoplasmic reticulum protein 44; KLF9, kruppel-like factor 9; HMGB2, high mobility group box 2; AIM2, absent in melanoma 2; Keap1, kelch-like ech-associated protein 1; ARE, antioxidant response element; FPN1, ferroportin 1; RGS12, role of G-protein signaling 12; HMG, high mobility group; TLR4, toll-like receptor 4; RAGE, receptor for advanced glycation end products; USP38, ubiquitin specific peptidase 38; DPP4, dipeptidyl peptidase 4; NOX1, NADPH oxidase 1; BNIP3, BCL2/adenovirus E1B 19 kDa protein-interacting protein 3; mtROS, mitochondrial reactive oxygen species; TfR-1, transferrin receptor 1; TRPM7, transient receptor potential melastatin 7; PI3K, phosphoinositide 3-kinase; ATF3, activating transcription factor 3; SOD-2, superoxide dismutase 2; XCT, cystine/glutamate antiporter; Akt, protein kinase B; mTOR, mechanistic target of rapamycin; MEF2D, myocyte enhancer factor 2D; NF-κB, nuclear factor kappa B; GSK3β, glycogen synthase kinase 3 beta; NEAT1, nuclear paraspeckle assembly transcript 1; GSDM, gasdermin; MyD88, myeloid differentiation primary response 88; IGFBP2, insulin-like growth factor binding protein 2; M2AChR, muscarinic acetylcholine receptor M2; OGDHL, oxoglutarate dehydrogenase-like; NRVMs, neonatal rat ventricular myocytes; AC16, AC16 human cardiomyocyte cell line; HL-1, HL-1 cardiomyocyte cell line; CMs, cardiomyocytes; NMVCs, neonatal mouse ventricular cardiomyocytes; EC, endothelial cell; H9c2, H9c2(2-1) rat cardiomyoblast cell line.
